# The Chronic Pains of the Chest

**Published:** 1912-07

**Authors:** Jno. Prather

**Affiliations:** Ft. Davis, Ala.


### THE CHRONIC PAINS OF THE CHEST.

By Dr. Jno. Prather, Ft. Davis, Ala.

Probably there is nothing which gives the young practitioner more trouble than pain in the chest among his patients. He soon learns to stop the acute pains but the chronic ones give even the older doctors trouble in treating them.

Some success is attained, however, by careful and repeated examinations of your patients and an honest endeavor to ascertain and relieve the cause. The patient expects relief, and this must be given as in acute pain, and the cause, or more often, causes found and relieved as soon as possible.

To find the cause, a history of the case, inquiry into the habits, and physical examination are necessary. If you first see the sufferer in the drug store or by the roadside, give temporary relief and insist on a visit to the office a few days later. If on first physical examination you cannot find the cause, try to diagnose by elimination of the most frequent causes in their order. In this locality they would be malaria, influenza, hookworms, rheumatism, rheumatic or gouty diatheses, and habit-forming drugs.

The drugs after salicylates and opiates to use on first seeing patient are nitrates, laxatives and quinine.

Alcohol after a spree or from too frequent use gives a pain similar to other things which upset the stomach. The pain of the opium user who has no serious trouble to cause other pains is usually referred to the sternum, but they are liable to describe it as being elsewhere. That from tobacco depends on the length of time used and the form. Chronic snuff users and cigarette smokers complain of a pain from epigastrium upward. The first is probably caused from swallowing the saliva and the latter from the resulting chronic bronchitis. The pain from the use of tobacco after the tobacco has affected the walls of the arteries and raised the blood pressure extends over the upper sternum, to the right side of the neck and to the left infraclavicular space. Every physician is familiar with the sound of a tobacco heart with its snapping second aortic sound and frequent mitral regurgitation. The pain from coffee is similar, but is even more liable to extend to the left infraclavicular space than to the right side of the neck.

Improper foods, such as sticky breads, hard fried meats, sweet potatoes, molasses, cabbage, and improperly cooked foods of many kinds, are responsible for a large number of cases. Most or all these patients have more than one cause for their pain. I cannot put too great stress on the foolishness of the promiscuous habit of putting these people on stomachics instead of giving them cooking lessons and the right kind of diet.

Many infections leave a chronic pain in this region and it is sometimes difficult to make a patient give a clear history of he ones he has been subject to in the last few months.

Suppurating cavities in distant regions may be at fault, either by metastasis to some thoracic region or reflexly, or from absorption of toxins. Pericarditis, endocarditis, pleurisy, and abscesses are among those by metastasis. Some of these will probably be found to be gonorrheal and it is well to include generative organs in your examination. Right here I will mention that some of the forms of rheumatism yield to serum therapy as well as protatitis in the male and peritonitis in the female.

Malaria is so persistent in this section that some of my neighboring practitioners complain that it looks sometimes as though we were becoming mere dispensers of calomel and quinine, but the fact remains that we have to treat most of our patients for malaria in addition to their other troubles or fail to cure them. In the acute attack the pain is everywhere, but in the most chronic forms the neck, shoulders and lower half of left thorax seem to bear the brunt of the pain. In the still more chronic forms the pain becomes neuralgic. This is a form that confuses us with the probability of its being rheumatism. The tender and enlarged spleen is a very persistent symptom and we are often called in during the winter to a case described as pneumonia which is an acute exacerbation of the last summer's malaria, the spleen inflamed and the lower half of the left thorax very painful with each respiration. In a few cases this is on the right side.

In winter months influenza seems to take precedence over malaria, but you have to make your diagnosis between it and malaria very cautiously.

The pains suffered by the hookworm patient are, so far as the chest is concerned, in the epigastrium, sternum, and right hypochondriac and mammary regions. This latter with me is diagnosis when the abdomen just to the right of the umbilicus is tender and there is history of infection, I treat them all. I com very near treatment them all for hookworms anyway.

In connection with hookworms should be mentioned anaemis, which sometimes affects the unfortunates like pseudo-locomotor ataxia. Whether the pain in the upper sternal region and neck in some of these cases is due to the thinness of the blood roaring through the common carotids or not, I doubt, but as soon as the blood gets thicker the pain stops.

In an older person with chronic rheumatism or gout and associated as it often is with arteriosclerosis, the pain in this region can be relieved more easily than you would think for. Give a shot-gun prescription for gout and rheumatism and dioscerea villosa, a drug I mention later. The pain will be constantly returning but will become more tractable each time, with this treatment. When you find one of these patients with painful chest and no evidence of arteriosclerosis, stop the dioscerea after the first week and give digitalis, not for the pain, however.

The pain in this region accompanying tuberculosis is usually not severe unless there is excessive coughing, or pleurisy. After the infection has spread to a number of thoracic tissues, the diagnosis is easier but too late to allow you to do more than relieve with the less weakening sedatives.

As lobar pneumonia, involving the surface of the lung is accompanied by localised pleurisy, we find a certain number of patients with old pleural adhesions which are more or less painful, but generally after a cold or exposure. This is one time that your treatment is assisted by blistering over the painful area.

The history is of some value in regard to syphilis, but among the negroes, a few symptoms are sufficient to warrant specific treatment. As painful chests are a prominent symptom among this class of patients and they thrive on mercury and iodids, it is well to try to relieve the symptoms with a few weeks' treatment along this line, after having eliminated other possible causes of the pain, without complete success.

The pain of the hysterical or neurasthenic is beyond the ordinary practitioner and these patients usually become dopers unless treated outside the home.

The patient who attributes an intercostal pain to a wrench, blow, strain, or fall, is to be looked upon with suspicion, and a further cause should be looked for, especially malaria. The pains from old wounds return again and again and the adhesions would be hard to break up in this part of the body. There will be observed some pleural adhesions from blows on the chest but I have never seen any of these become painful.

Pain from bronchitis is usually over the upper sternum, but in certain chronic forms, as that from smoking, it is referred to the lower sternum and epigastrium.

The lungs themselves are not usually the cause of the more chronic pains, but the tissues adjacent. In certain trades the lungs get filled with dust or lint and feel uncomfortable as they do in emphysema and asthma, but no real pain always accompany these affections.

The organs most frequently at fault when a pain appears in the mediastinum are the air, blood, and food passages, making a careful examination necessary before ascribing it to a habit or infection. I have never in my own practice diagnosed any tumors right here, where I was sure of my diagnosis, except a few anuerisms. The cardiac pains are too many to detail but are not so frequent after all as the arterial. Then, too the clinician can make a surer diagnosis here than he can with the cardiac troubles unless the latter are organic. The nitrates have long been standard and they are used promiscuously. I never heard of them curing and the effect is so transient that they are only of service except in some acute arterial spasm when you wish a quick effect.

Most of you have become tired of iodids to help you with these hardened arterial and cardiac tissues. In treating pain in this area and farther up toward the neck, whether caused by coffee, tobacco, rheumatism, arteriosclerosis, anuerism of one of the large vessels and other painful or choking sensations, I have been accustomed to use an old-fashioned drug which the various books claim to do so many things that I will just tell you what I have seen it do or assist other drugs to do. Well, it is a laxative and in that respect is superior to cascara. It is diuretic and pleasantly so. It is expectorant, leaving the mouth moist. It is slightly diaphoretic. In large doses, an emetic. It relieves intestinal indigestion or so-called biliousness by promoting a flow of bile from the gall bladder or liver. I have seen it give permanent relief in inflammation of the gall bladder apparently from non-suppurative gall stones and the books recommend it for this. Some relief in suppurative cases. It is an antispasmodic as its name of cramp bark would indicate, being especially valuable in colic of any part of the alimentary tract. But better than all these other effects, it will reduce blood pressure and help relieve these cardiac and arterial spasms. This drug has effect in a few minutes and will keep up the effect of your previously administered nitroglycerin and in the less acute forms it is better than anything else. I usually, but not all the time, give iodids with it. When you put it in solution in a prescription you had better put a little creosote in it to prevent fermentation. In a fatal case of thoracic aneurism with bulging chest wall and strutting carotids this drug lessened the pain and calmed down the carotids to some extent. I have on hand an old negro about sixty-five years old who came for treatment about a year ago with a very painful anuerism of the right common carotid. He has not required any treatment for it in some time and he tells me his neck never hurs him except after a little hard, straining labor. It is now about half the size it was a year ago. During the last year and a half I have seen symptoms of thoracic anuerism disappear with this treatment and they now have no pain but they cut down their coffee and tobacco at the same time and I will not be too certain in my diagnosis of anueris in more than two of these cases. The number of coffee and tobacco users who have been relieved of their pain with this drug is surprising even to myself. They constantly return to their habits and the pain returns but does not go even when they quit, but they have to take their dioscerea nearly every time to get rid of it.

The pains over the mediastinum, which seem to be esophageal, are usually reflected from the stomach unless there is an injury or malformation of the esophagus. There is one very important exception and that is the esophageal burning in beginning pellagra. When the mouth is sore, epigastrium tender, there is diarrhoea or dysentery, and the limbs ache, it is well to look for skin symptoms soon. I made a tentative diagnosis with these symptoms once and was later justified.

(I omit here the discussion of pains in the chest from gynecological troubles and the liability of these patients becoming dope fiends, as the paper was originally read.)

There is one very frequent complaint among women who come to the office, in connection with pregnancy. The pain extends above and below the epigastrium and often appears before any vomiting, nausea or swollen extremities. Generally alkaline drinks and treatment for the albuminuria relieve this for a few weeks or as long as the albuminuria can be kept down.

In older people the pains accompanying chronic renal troubles are usually caused in the chest by the resulting hardening of the blood vessels and should be treated as such.

The milder hepatic troubles causing pain can usually be relieved as stated before with dioscerea. But here as elsewhere, free purgation should precede the systematic administration of this drug for chronic troubles. Fractional doses of calomel for four days are often of assistance. The dose of the Fluidextract of Dioscerea is from thirty to sixty minims. For constipation, a teaspoonful every morning to act the next morning. For griping pain, one to two teaspoonfuls, effect in ten to fifteen minutes. To lower blood pressure, about four thirty to sixty minim doses a day. It will wear out in thirty days given for any effect and it is necessary to hold up a week or so and start over again. Two of my cases with pain over hepatic area were from extreme right end of liver separated from its attachment to diaphragm.

One this year and the other with history of injury ten years ago. Their pain is not constant.

July 10th, 1912.—The above paper was withheld from publication because I had no authority but my own for the fact that the Flext. of Dioscerea Villosa would reduce blood pressure. But I have been using it rationally on that basis now for another year with even better results and one case in the last two weeks has proved my point even more conclusively.

Negro, male, aged thirty, gonorrhea three years ago with slightly increasing uneasiness in breathing up to a few weeks ago when his breath became very short. He came ten days ago for treatment and physical examination showed an aortic stenosis and insufficiency and a pulmonary stenosis with a hard snap on closure. The carotids were pulsating as were the arteries on the temples and his breath was very short. He had the cough and sputum characteristic of pulmonary edema from broken cardiac compensation. I knew that digitalis was contra-indicated and as I have never used strophanthus or convalaria, so was in a quandary. I gave him Flext. Dioscerea Villosa in teaspoonful doses four times a day with direction to stop immediately if he did not improve and to return for other treatment; to rest unless his breathing was good. He improved from the first dose and did field work regularly, bad weather permitting. His uneasiness returned last night after taking the last dose yesterday morning. Returned to office to-night. The murmurs were still present but were softer. The hard pulmonary snap had disappeared and the carotid and other pulsations were less prominent, and he said they had been even less while under the effect of the medicine. I gave him another bottle and now he is taking it.

During the year the carotid anuerism case has been getting along splendidly without treatment. A lady with coffee heart has been having to take a few doses of wild yam occasionally. One of the young colored women to whom I have been giving it off and on for two years for snuff pains still uses snuff and comes for her wild yam to enable her to continue to do so. I have been trying to get her to go to another doctor as she will not quit the snuff. Another young colored girl whose chest pain it relieved for a few weeks two years ago has at last developed sufficiently acute appendicitis to diagnose and is now convalescing. The relief from the wild yam at the time was probably from the benefit to the griping and constipation and not so much from a snuff pain as I thought at the time. I have recently used it one hour after aloes to prevent gripin. The aloes were used as an emenegog and I did not wish to use hyoscyamus or belladonna.

